# Supplementary material for: The nucleosomal acidic patch relieves auto-inhibition by the ISWI remodeler SNF2h
Source: eLife. 2018 Apr 17;7:e35322. doi: 10.7554/eLife.35322 (PMC5976439; doi:10.7554/eLife.35322)
Supplement: Transparent reporting form [file elife-35322-transrepform.docx]

***eLife’s* transparent reporting form**

We encourage authors to provide detailed information *within their submission* to facilitate the interpretation and replication of experiments. If you have any questions, please contact us: [editorial@elifesciences.org](mailto:editorial@elifesciences.org).

**Sample-size estimation**

- You should state whether an appropriate sample size was computed when the study was being designed
- You should state the statistical method of sample size computation and any required assumptions
- If no explicit power analysis was used, you should describe how you decided what sample (replicate) size (number) to use

Please outline where this information can be found within the submission (e.g., page numbers or figure legends), or explain why this information doesn’t apply to your submission:

For the quantitative biochemistry assays, the minimum number of replicates used is 3 (N=3). Replicate information is found in figure legends. For single-molecule experiments, we started with the sample size used in a previous study (see PMC2835771) and confirmed the appropriateness of this sample size by doubling the number of molecules included in the analysis and observing no change to measured values within error (i.e. we used a subsampling estimate for sample size computation). Sample size for smFRET is given in the Methods on page 20.

**Replicates**

- You should report how often each experiment was performed
- You should include a definition of biological versus technical replication
- The data obtained should be provided and sufficient information should be provided to indicate the number of independent biological and/or technical replicates
- If you encountered any outliers, you should describe how these were handled
- Criteria for exclusion/inclusion of data should be clearly stated
- High-throughput sequence data should be uploaded before submission, with a private link for reviewers provided (these are available from both GEO and ArrayExpress)

Please outline where this information can be found within the submission (e.g., page numbers or figure legends), or explain why this information doesn’t apply to your submission:

For ensemble data: details on replicate experiments are reported on each figure legend. Replicate data were obtained from ≥ 3 independent experiments. For single-molecule: as noted on page 20, experiments were repeated independently at least 3 (typically 5 to 7) times. The Methods on page 20 also detail exclusion criteria used in addition to standard criteria for exclusion of FRET data (e.g. data where donor and acceptor dye intensities were not anti-correlated). A single biological replicate was performed of the cross linking experiment.

**Statistical reporting**

- Statistical analysis methods should be described and justified
- Raw data should be presented in figures whenever informative to do so (typically when N per group is less than 10)
- For each experiment, you should identify the statistical tests used, exact values of N, definitions of center, methods of multiple test correction, and dispersion and precision measures (e.g., mean, median, SD, SEM, confidence intervals; and, for the major substantive results, a measure of effect size (e.g., Pearson's r, Cohen's d)
- Report exact p-values wherever possible alongside the summary statistics and 95% confidence intervals. These should be reported for all key questions and not only when the p-value is less than 0.05.

Information on replicates and statistical analysis described in relevant figure legends and in the methods section. Errors for ensemble biochemistry are reported as standard error of the mean (SEM). For single-molecule: statistical and error estimation methods are described in the methods section (see page 20). Errors were bootstrapped (see page 20). For crosslinking-MS the reliability of the crosslinked peptide identifications was assessed using a decoy database approach to model the probability of a random match. The size of the decoy database and modeling approach used is outlined in the experimental methods and in references therein.  The overall false discovery rate for the dataset reported according to the selection criteria outlined is reported.

Please outline where this information can be found within the submission (e.g., page numbers or figure legends), or explain why this information doesn’t apply to your submission:

(For large datasets, or papers with a very large number of statistical tests, you may upload a single table file with tests, Ns, etc., with reference to page numbers in the manuscript.)

**Additional data files (“source data”)**

- We encourage you to upload relevant additional data files, such as numerical data that are represented as a graph in a figure, or as a summary table
- Where provided, these should be in the most useful format, and they can be uploaded as “Source data” files linked to a main figure or table
- Include model definition files including the full list of parameters used
- Include code used for data analysis (e.g., R, MatLab)
- Avoid stating that data files are “available upon request”

Please indicate the figures or tables for which source data files have been provided:

Relevant source data is provided in the main and supplemental figures. Crosslinked residue pair identification along with number of spectral counts per identification are reported in Supplemental File 1, as well as in a web resource with links to annotated product ion spectra (see Experimental Methods).  Raw mass spectrometry files are available on the Massive server (UCSD). Code used for the analysis of smFRET data can be found at the following link, which is also cited in the main text. <https://github.com/stephlj/Traces>
